# OsANN4 modulates ROS production and mediates Ca^2+^ influx in response to ABA

**DOI:** 10.1186/s12870-021-03248-3

**Published:** 2021-10-18

**Authors:** Qian Zhang, Tao Song, Can Guan, Yingjie Gao, Jianchao Ma, Xiangyang Gu, Zhiguang Qi, Xiaoji Wang, Zhengge Zhu

**Affiliations:** Hebei Key Laboratory of Molecular and Cellular Biology, Key Laboratory of Molecular and Cellular Biology of the Ministry of Education, College of Life Science, Hebei Normal University, Hebei Collaboration Innovation Center for Cell Signaling, Shijiazhuang, 050024 China

**Keywords:** Annexin, Abscisic acid, ROS, Ca^2+^ influx, Calcium-dependent protein kinase

## Abstract

**Background:**

Plant annexins are calcium- and lipid-binding proteins that have multiple functions, and a significant amount of research on plant annexins has been reported in recent years. However, the functions of annexins in diverse biological processes in rice are largely unclear.

**Results:**

Herein, we report that OsANN4, a calcium-binding rice annexin protein, was induced by abscisic acid (ABA). Under ABA treatment, the plants in which OsANN4 was knocked down by RNA interference showed some visible phenotypic changes compared to the wild type, such as a lower rooting rate and shorter shoot and root lengths. Moreover, the superoxide dismutase (SOD) and catalase (CAT) activities of the RNAi lines were significantly lower and further resulted in higher accumulation of O_2_^.-^ and H_2_O_2_ than those of the wild-type. A Non-invasive Micro-test Technology (NMT) assay showed that ABA-induced net Ca^2+^ influx was inhibited in *OsANN4* knockdown plants. Interestingly, the phenotypic differences caused by ABA were eliminated in the presence of LaCl_3_ (Ca^2+^ channel inhibitor). Apart from this, we demonstrated that OsCDPK24 interacted with and phosphorylated OsANN4. When the phosphorylated serine residue of OsANN4 was substituted by alanine, the interaction between OsANN4 and OsCDPK24 was still observed, however, both the conformation of OsANN4 and its binding activity with Ca^2+^ might be changed.

**Conclusions:**

*OsANN4* plays a crucial role in the ABA response, partially by modulating ROS production, mediating Ca^2+^ influx or interacting with OsCDPK24.

**Supplementary Information:**

The online version contains supplementary material available at 10.1186/s12870-021-03248-3.

## Background

Abscisic acid (ABA), a well-known long-distance signaling molecule utilized for communication between plant roots and shoots under water-deficient conditions, is also considered a hormone that plays a critical role in abiotic stress tolerance in plants [1, 2]. Recently, many mediators of ABA signaling, such as ABA receptors [3, 4] and targets of ABA receptors [5, 6], have been characterized. Since the identification of the steroidogenic regulatory protein (StAR)-related lipid-transfer (START) domain as a candidate ABA receptor, pyrabactin resistance 1 (PYR1) and PYR1-like 1-13 (PYL1-PYL13) have been considered key components of the core ABA signaling pathway [7, 8]. ABA functions as an important phytohormone to regulate the expression of many genes, leading to complex physiological and metabolic responses that enable plants to confer tolerance to abiotic stress [9, 10]. Increasing evidence shows that ABA-enhanced abiotic stress tolerance might be associated with the induction of antioxidant defense systems [11, 12]. Reactive oxygen species (ROS), as an intermediate component, play an essential role in ABA-induced antioxidant defense [13, 14]. Low concentrations of ROS can be used as signaling molecules to regulate the response of plants to ABA signals [15, 16]. The massive accumulation of ROS leads to redox imbalance, causing protein, DNA and lipid damage and even the death of plants [17, 18]. To ensure the proper function and survival of plant cells, it is very important to rapidly eliminate the massive ROS. The defense system of enzymatic scavengers, including catalase (CAT), superoxide dismutase (SOD), ascorbate peroxidase (APX) and glutathione reductase (GR), plays an essential role in the elimination of ROS [19, 20].

Ca^2+^ signals also function as vital signaling molecules in the ABA response [13, 21]; for instance, the activation of Ca^2+^-permeable cation channels leads to an increase in cytosolic free Ca^2+^ concentration ([Ca^2+^]_cyt_), further enhances the activity of S-type anion channels and promotes stomatal closure [22, 23]. The recruitment of ABA receptors to the membrane is controlled by Ca^2+^, and Ca^2+^-dependent protein kinases (CDPKs) as well as calcineurin B-like proteins/CBL interacting protein kinases (CBL/CIPKs) are recognized links between Ca^2+^ signaling and ABA responses [24]. CDPKs are serine/threonine protein kinases that function as one of the best characterized Ca^2+^ sensors in plants [25]. Two *Arabidopsis thaliana* CDPKs, *AtCPK3* and *AtCPK6*, are positive regulators in ABA regulation of Ca^2+^-permeable channels and Ca^2+^ activation of S-type anion channels [26]. Genetic evidence has shown that *AtCPK4* and *AtCPK11*, as important components, function in CDPK/calcium-mediated ABA signaling processes, including seed germination, seedling growth and guard cell regulation [27]. *AtCPK10* functions in the response to drought stress by modulating ABA and Ca^2+^-mediated stomatal movements [28].

Annexins belong to an evolutionarily conserved multi-gene protein superfamily comprising Ca^2+^-dependent phospholipid-binding proteins [29, 30]. Plant annexins are reportedly tissue-specific and play an important role in plant stress responses [31–34]. The alfalfa (*Medidago sativa*) annexin gene *MsANN2* was first reported to be activated by drought and ABA [35]. Rice annexin *OsANN3* regulates the drought stress response in an ABA-dependent pathway [36]. Both *OsANN1* and *OsANN10* confer resistance to abiotic stress in rice by modulating ROS and/or lipid peroxidation levels [37, 38]. To date, *AtANN1* is the most widely studied plant annexin, and it functions in *Arabidopsis* in response to a variety of abiotic stresses (such as osmotic stress, drought, salt, ABA, cold, heat and hydrogen peroxide) [39–44]. In response to osmotic stress, high salinity, ABA, cold and heat stress, *AtANN1* is tightly associated with the regulation of Ca^2+^ [39, 41–43]. However, as a redox sensor, AtANN1 has peroxidase activity and is involved in the response to drought stress by regulating ROS [40, 45]. In addition, as a linker of ROS- and [Ca^2+^]_cyt_-driven signals [46], AtANN1 could participate in H_2_O_2_-activated Ca^2+^ flux. Under 10 mM H_2_O_2_ treatment, Col-0 showed a sustained elevation of net Ca^2+^ influx and [Ca^2+^]_cyt_ at a certain time, but in the roots and root epidermal protoplasts of the *AtANN1* knockout mutant, they were aberrant [44].

Sequence analysis revealed that annexin may have a posttranscriptional modification site, such as a phosphorylation site, which may be a substrate for protein kinases [47]. Evidence obtained using a tandem affinity purification approach to identify protein complexes suggests that annexin may interact with various kinases, including receptor-like kinase, sterile-20 (Ste20)-like kinase, calcium/calmodulin-dependent protein kinase and casein kinase [48]. Quantitative phosphorylation proteomics identification results showed that AtANN1 can be phosphorylated by AtSnRK2s, which are the central component of the ABA signaling pathway [49]. A recent study showed that protein kinase open stomatal 1 (OST1/SnRK2.6) phosphorylates AtANN1 under cold stress and increases its Ca^2+^ transport capacity, which is essential for regulating the freezing tolerance of *Arabidopsis* [42]. As a substrate phosphorylated by the protein kinase SOS2, AtANN4 plays a vital role in salt stress-induced calcium signaling, which activates the SOS pathway in *Arabidopsis* [50]. Mu et al. demonstrated that the protein phosphatase GhDsPTP3a dephosphorylates GhANN8b and that GhDsPTP3a-GhANN8b participates in the response of cotton to salt stress by regulating the export of Na^+^ [51].

In this study, a putative rice annexin, OsANN4, was characterized, and its functions in maintaining the ROS balance and response to ABA were explored. We found that OsANN4 is a calcium-binding protein and related to the redox balance, as well as a substrate of the protein kinase OsCDPK24. Our results suggested that OsANN4 responds to ABA by modulating ROS and Ca^2+^ signals or interacting with OsCDPK24 in rice.

## Results

### *OsANN4*, is responsive to ABA

**Fig. 1:**
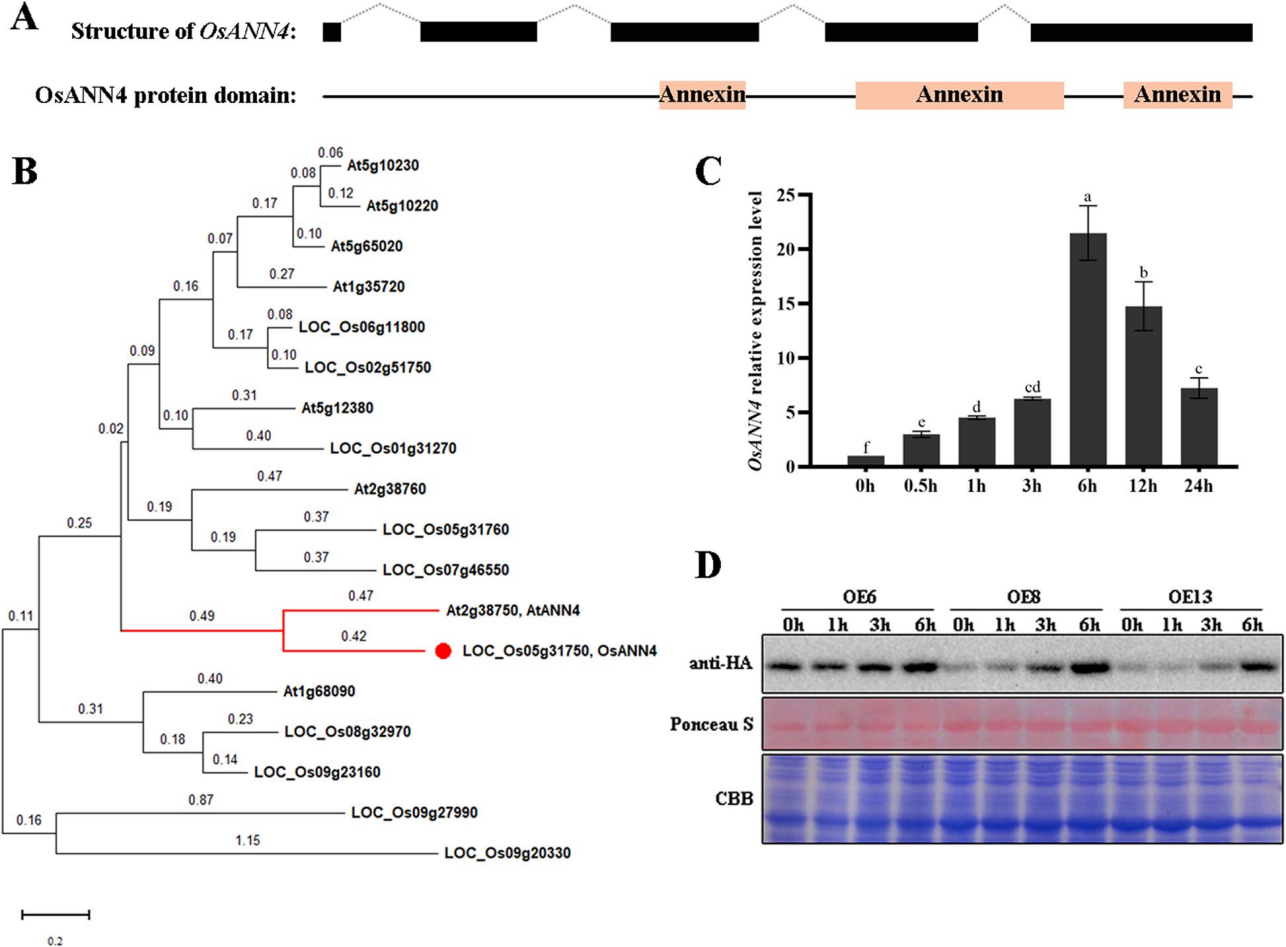
*OsANN4* responds to exogenous abscisic acid in rice. **a** Schematic diagram of the gene structure and protein domain of OsANN4. Exons, introns and protein domains are indicated by black boxes and dotted lines between black boxes and orange boxes, respectively. **b** Phylogenic tree of OsANN4 and the Arabidopsis ortholog AtANNs. The phylogenetic trees were constructed with Mega X using the maximum likelihood method. OsANN4 is marked with a red dot. **c** *OsANN4* transcript expression levels under ABA treatment. *OsACTIN1* was used as the internal control. **d** Immunoprecipitation of OsANN4 in *OsANN4*-overexpressing lines treated with ABA for 1 h, 3 h and 6 h. Values represent the means ± SD from three independent repeats, and different letters indicate significant differences (one-way ANOVA, *P*<0.05)

Based on bioinformatics, there are ten putative annexin genes in the rice genome, and we previously reported that *OsANN1*, *OsANN3* and *OsANN10* are involved in the response to heat, drought and osmotic stress [36–38]. To obtain a more comprehensive understanding of the function of rice annexin, a putative annexin family gene (LOC_Os05g31750) was cloned from *Oryza sativa L. spp. Japonica*, whose genomic sequence consists of 5 exons and 4 introns, and the open reading frame encodes a protein of 320 amino acids. A sequence search based on the NCBI database revealed that the protein contains three annexin domain architectures, so-called annexin repeats, which comprise segments of 33, 58, and 49 amino acid residues (Fig. 1a). Phylogenetic analysis showed that LOC_Os05g31750 is an ortholog of AtANN4, so it was named OsANN4 (Fig. 1b).

We treated 7-d-old wild-type seedlings with 10 μM ABA and examined the expression of *OsANN4* at 0 h, 0.5 h, 1 h, 3 h, 6 h, 12 h and 24 h. The results showed that the expression of *OsANN4* was induced by ABA treatment and reached the highest expression level after treatment for 6 h (Fig. 1c). To test whether ABA can induce the expression of OsANN4 at the protein level, the Ubi_pro_::*OsANN4*-HA vector was transformed into rice, and 15 overexpressed *OsANN4* (*OsANN4*-OE) lines were obtained. Quantitative real-time PCR (qRT-PCR) results showed that the expression level of *OsANN4* in all overexpressed lines was higher than that of WT, and 6, 8 and 13 lines were selected for follow-up experiments (Figure S1a). We also treated the overexpression lines with 10 μM ABA, and the results showed that the OsANN4 protein gradually increased after ABA treatment (Fig. 1d), indicating that *OsANN4* may respond to exogenous abscisic acid.

**Fig. 2:**
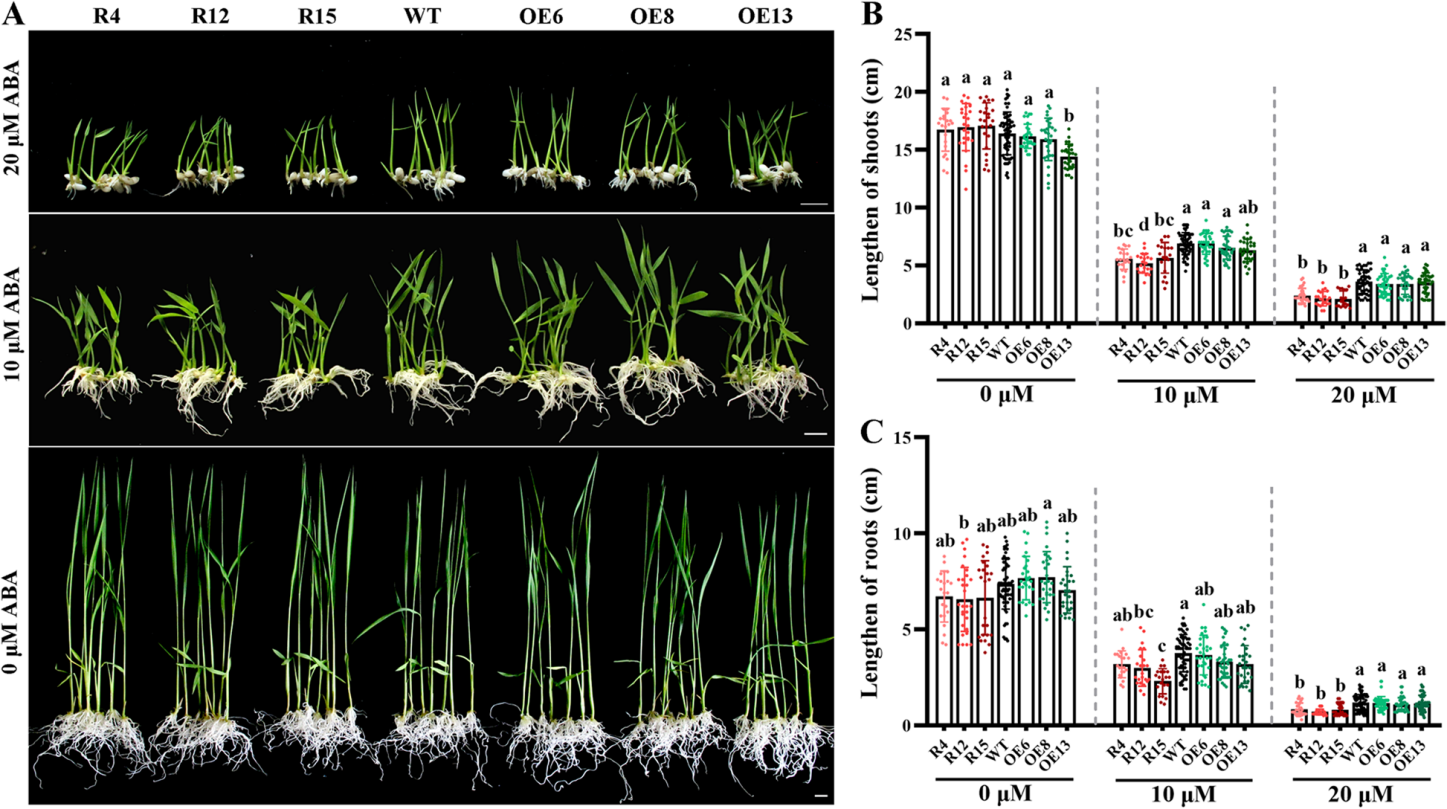
Growth analysis at the seedlings stage. **a** WT and *OsANN4* transgenic lines were grown for 18 d in 1/2 MS medium with 0, 10 or 20 μM ABA. Scale bars are 1 cm. The figure shows the representative results of five replicates with T2 generation rice. Statistical analysis of shoot height (**b**) and root length (**c**) at 18 d. Values represent the mean ± SD of plants for three independent experiments (n≥21). Statistical significance was determined by one-way ANOVA, *P*<0.05

To identify the function of *OsANN4* in the ABA signal response, we introduced *OsANN4*-RNAi into rice and obtained *OsANN4* knockdown plants (*OsANN4*-RNAi). QRT-PCR analyses showed that *OsANN4* was downregulated in all *OsANN4*-RNAi lines (Figure S1b). Homozygous plants of RNAi lines (R4, R12 and R15) were used for further analysis. We planted seeds from WT, *OsANN4*-RNAi and *OsANN4*-OE lines on 1/2 Murashige and Skoog (MS) medium supplemented with 0 (as control), 10 and 20 μM ABA. After three days, the seeds of all lines on all media germinated (Figure S2a). However, with the presence of 10 or 20 μM ABA, the rooting rate of *OsANN4*-RNAi lines was significantly lower than that of WT and *OsANN4*-OE lines in the following days, and no differences were observed without ABA (Figure S2b-d). Moreover, *OsANN4*-RNAi plant growth was inhibited with ABA treatment (Fig. 2a), the shoot length and root length of *OsANN4*-RNAi plants were significantly lower than those of WT and *OsANN4*-OE plants in the presence of ABA, especially when 20 μM ABA was applied, and no differences were observed in all plants without exogenous ABA (Fig. 2b, c). The data indicated that *OsANN4* may play a crucial role in the response to ABA signals in rice.

### *OsANN4* modulates antioxidant enzyme activities and ROS production in response to ABA

**Fig. 3:**
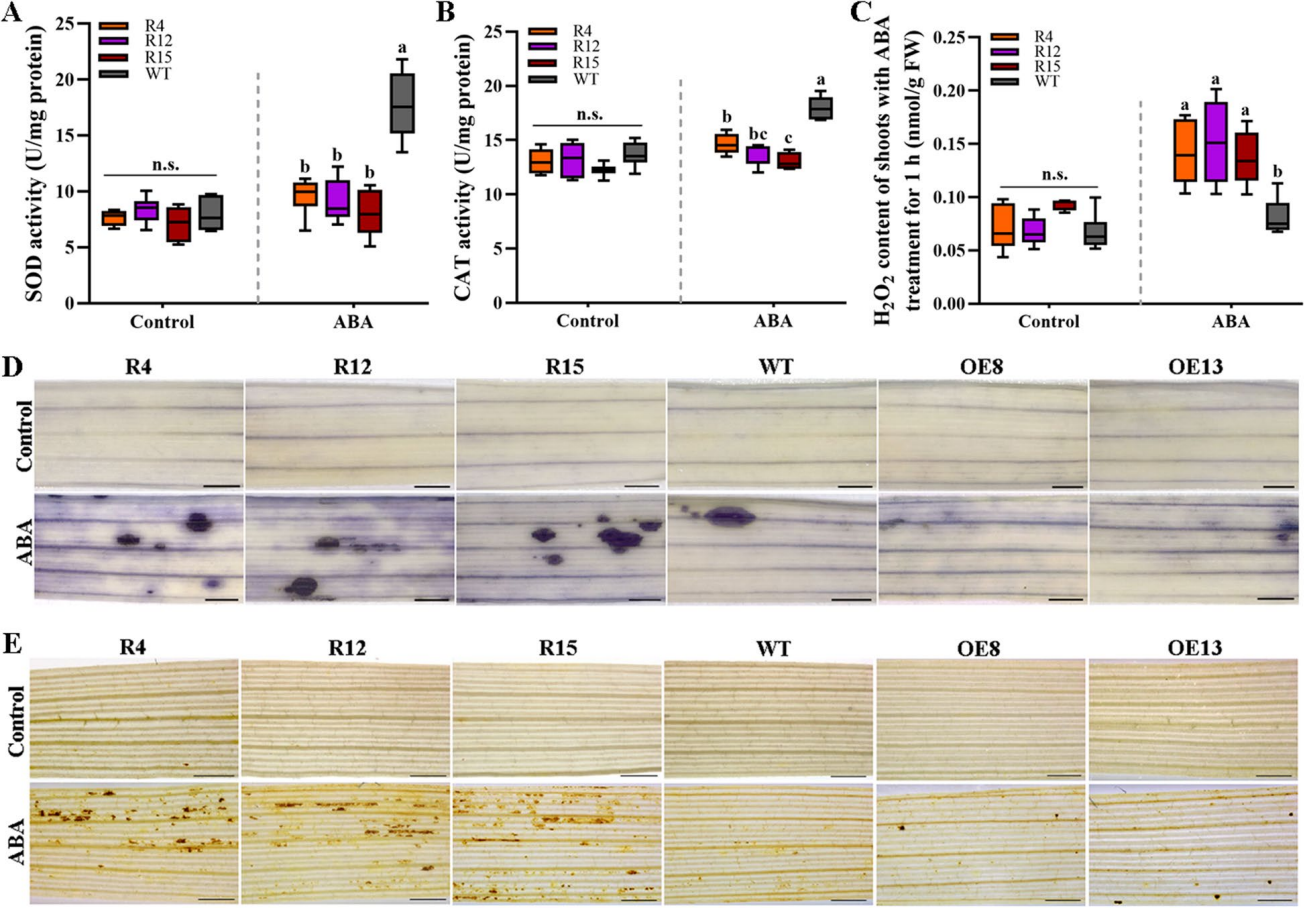
*OsANN4* modulates antioxidase activity and ROS products under ABA treatment. SOD activities (**a**) and CAT activities (**b**) in 7-d-old WT and *OsANN4* knockdown plants with or without 30 μM ABA treatment for 0.5 h. **c** The H_2_O_2_ content in the shoots of 7-d-old WT and *OsANN4* knockdown plants with or without 30 μM ABA treatment for 1 h. NBT staining for O_2_^.-^ (**d**) and DAB staining for H_2_O_2_ (**e**) in situ in the leaves of WT and *OsANN4* knockdown and overexpressed plants with or without ABA treatment. Scale bars for images (**d**) and (**e**) are 1 mm. Values represent the mean ± SD from three independent experiments. Statistical significance was determined by one-way ANOVA, *P*<0.05

More evidence suggests that ABA-enhanced stress tolerance is associated with the induction of the antioxidant defense system to protect plant cells against oxidative damage [12, 52]. SOD and CAT may protect plant cells from oxidative damage by eliminating ROS, which are important signaling molecules in the ABA pathway. To assess the effect of knocking down *OsANN4* on antioxidant defense to ABA response, we detected the activities of SOD and CAT of 7-d-old seedlings without or with 30 μM ABA treatment for 0.5 h. The *OsANN4*-RNAi lines showed no significant difference in activities of both SOD and CAT relative to that of the WT plants without ABA treatment. After ABA treatment, SOD and CAT activities of *OsANN4*-RNAi lines were lower than those of the WT plants (Fig. 3a, b).

Next, the H_2_O_2_ content of 7-d-old rice seedlings was further detected with or without ABA treatment for 1 h. The results showed that H_2_O_2_ content was significantly higher in *OsANN4*-RNAi lines than in the WT (Fig. 3c). Next, the production of H_2_O_2_- and O_2_.- was detected in situ in 7-d-old seedlings with 3,3’-diaminobenzidine (DAB) and nitro-blue tetrazolium (NBT) staining, respectively, and no visible differences were observed in any plants without exogenous ABA treatment. However, the blue spots (reflecting O_2_^.-^ production) or brown spots (reflecting H_2_O_2_ production) in the mesophyll cells of *OsANN4*-RNAi lines were significantly increased compared with those of WT and *OsANN4*-OE plants when exogenous ABA was present (Fig. 3d, e). This indicated that the production of O_2_^.-^ and H_2_O_2_ was related to *OsANN4* expression under exogenous ABA treatment in rice.

### OsANN4 is a Ca^2+^-binding protein that is located on the cell periphery

**Fig. 4:**
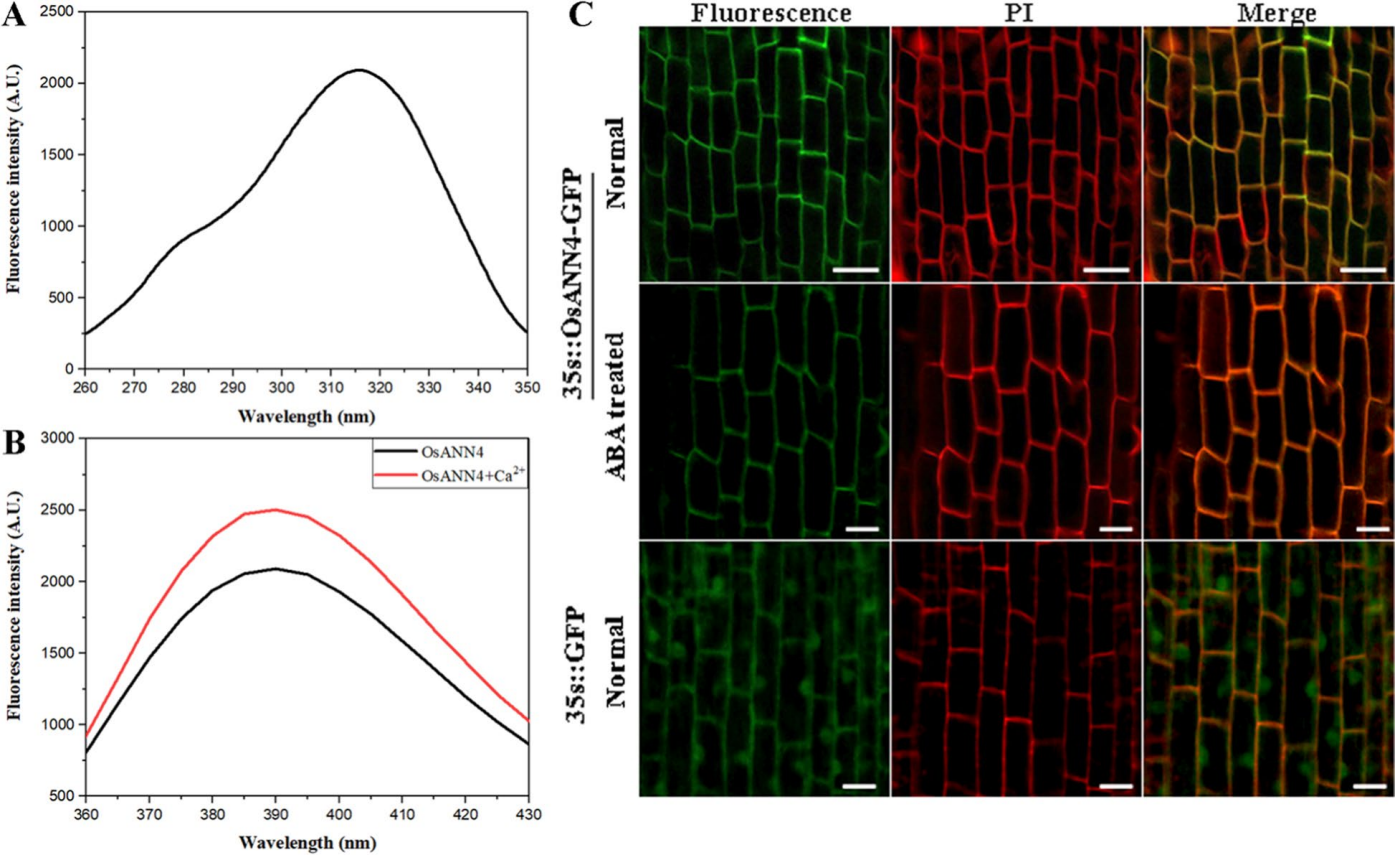
Analysis of the subcellular localization and Ca^2+^-binding characteristics of OsANN4. **a** Fluorescence excitation of the purified OsANN4-His protein. **b** Emission spectra of the purified OsANN4-His protein. **c** Subcellular localization of OsANN4-GFP in 3-d-old root tip cells, GFP was used as a control. Scale bars are 20 μm

Annexins are considered to be a class of proteins interacting with biological membranes in a calcium-dependent or calcium-independent manner. In this study, we detected the Ca^2+^-binding activity of OsANN4. The fluorescence level of OsANN4-His recombinant protein was determined by a fluorescence spectrophotometer. Upon excitation at 315 nm (Fig. 4a), the fluorescence emission spectrum showed the maximum fluorescence wavelength (λ max) at 390 nm, where the fluorescence intensity reached approximately 2000 A.U.. The maximum fluorescence intensity of the OsANN4-His recombinant protein was measured again after the addition of 2 mM Ca^2+^. The results showed that the maximum fluorescence wavelength remained unchanged, whereas the fluorescence intensity changed (Fig. 4b), indicating that OsANN4 has Ca^2+^ binding activity and may further change the conformation of the protein.

Previous studies have shown that the subcellular localization of annexin may be altered due to environmental stimuli [37, 53]. To explore whether OsANN4 altered its subcellular localization with ABA treatment, the 35S_pro_::*OsANN4*-GFP vector was introduced into *Agrobacterium* EHA105 and then transformed into rice calli. Twenty-five independent transgenic lines were obtained, and 3 lines were used for further analyses. OsANN4-GFP signals were observed in the cell periphery through a confocal laser-scanning microscope, and GFP alone was ubiquitously expressed in the cell. However, the signal of OsANN4-GFP could not be altered when 10 μM ABA was present (Fig. 4c).

### OsANN4 may mediate ABA-induced Ca^2+^ flux

**Fig. 5:**
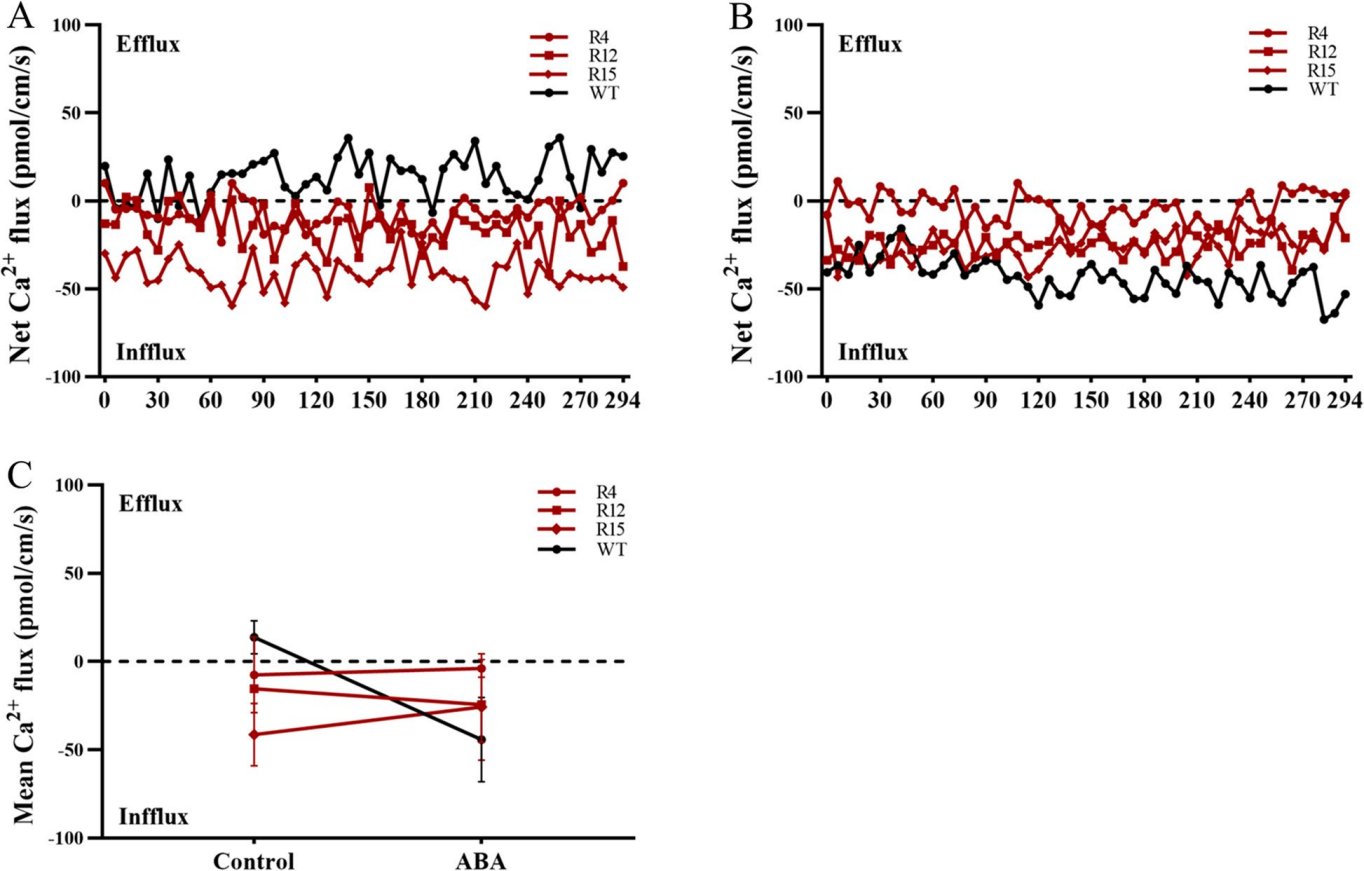
Knockdown of *OsANN4* inhibits ABA-induced Ca^2+^ influx in rice root tips. **a** The net Ca^2+^ flux was examined with NMT under normal conditions. **b** The net Ca^2+^ flux was examined with NMT under ABA treatment. **c** The mean Ca^2+^ flux under normal conditions and ABA treatment. Values represent means ± SD from three independent repeats

Some AtANNs, such as AtANN1 and AtANN4, mediate stress-induced increases in [Ca^2+^]_cyt_ [41–44, 50, 54]. To test whether OsANN4 is involved in Ca^2+^ transients, we measured Ca^2+^ flux at the meristem zone of 3-d-old WT, RNAi and OE plants following a 0.5 h treatment with 30 μM ABA using Non-invasive Micro-testing Technology (NMT). Without exogenous ABA treatment, there was a weak efflux of Ca^2+^ in WT roots, and the Ca^2+^ in *OsANN4*-RNAi and *OsANN4*-OE roots was in an influx state (Fig. 5a). With ABA treatment, all lines showed an influx of Ca^2+^, and the influx rate of Ca^2+^ in *OsANN4*-RNAi plants was lower than those in WT and *OsANN4*-OE plants (Fig. 5b). Compared with untreated plants, the mean influx rate of extracellular Ca^2+^ in WT and *OsANN4*-OE roots increased significantly, while the mean influx rate of extracellular Ca^2+^ in *OsANN4*-RNAi roots did not change obviously (Fig. 5c). The data suggest that OsANN4 may mediate Ca^2+^ influx and be involved in the response to ABA.

**Fig. 6:**
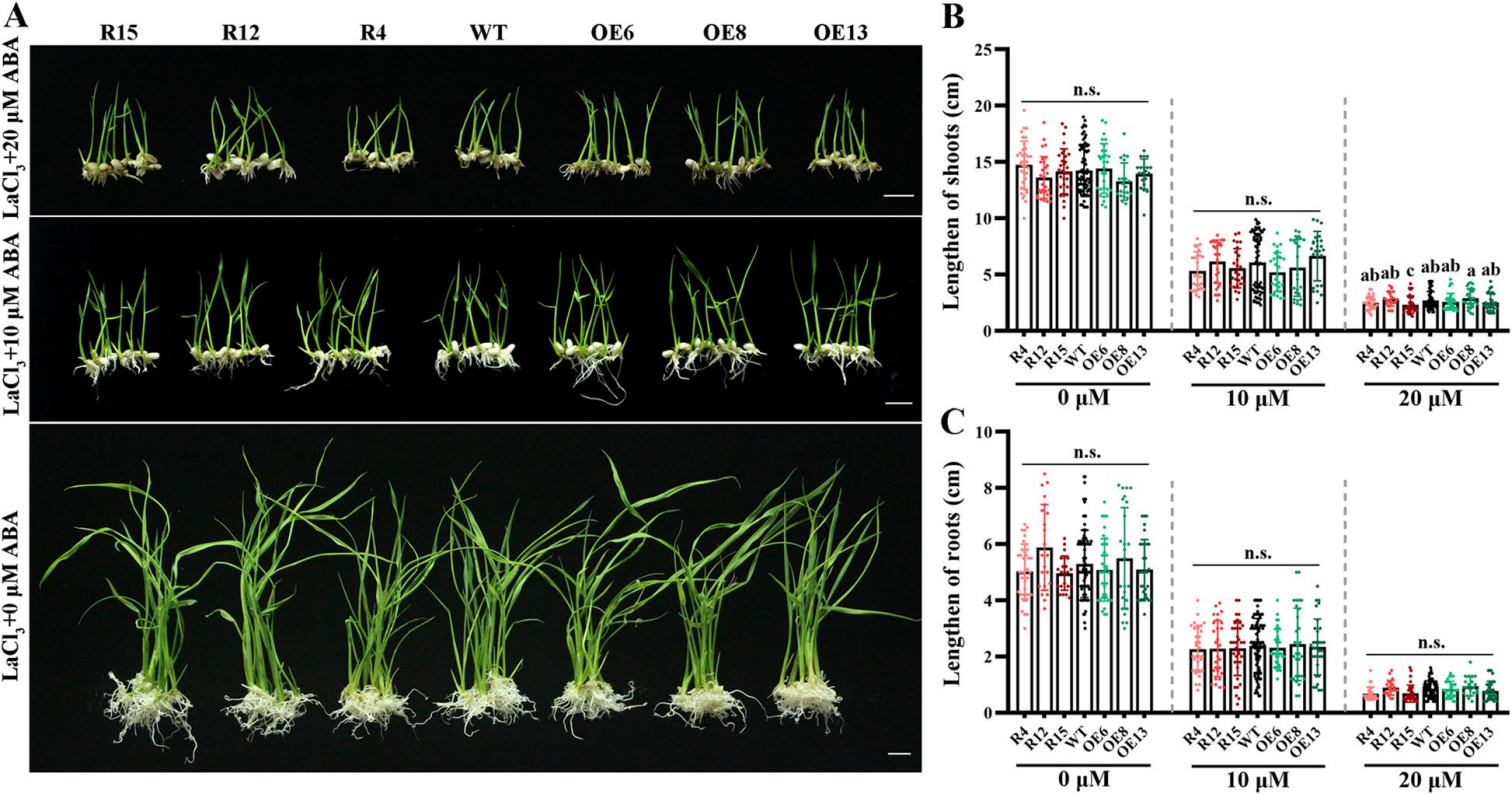
Growth analysis of seedling stages in the presence of LaCl_3_. **a** WT and *OsANN4* transgenic lines were grown for 18 d in 1/2 MS medium with 0, 10 or 20 μM ABA in the presence of LaCl_3_. Scale bars are 1 cm. The figure shows the representative results of five replicates with T2 generation rice. Statistical analysis of shoot height (**b**) and root length (**c**) at 18 d. Values represent the mean ± SD of plants for three independent experiments (n≥21). Statistical significance was determined by one-way ANOVA, *P*<0.05

To test whether OsANN4-mediated Ca^2+^ influx is important for the ABA response, we planted seeds from WT, *OsANN4*-RNAi and *OsANN4*-OE lines on 1/2 MS medium supplemented with 0, 10 and 20 μM ABA while adding 100 μM LaCl_3_. As a Ca^2+^ channel inhibitor, LaCl_3_ can inhibit the flux of Ca^2+^ between the apoplast and cytoplasm. More than 95% of seeds of all lines germinated in two days with or without ABA (Figure S3a). In the presence of LaCl_3_, the difference in the rooting rate caused by ABA was reduced (Figure S3b-d). After 18 d, whether 0, 10 or 20 μM ABA was added, there was no obvious phenotypic difference among all the plants (Fig. 6a). The statistical results indicated that ABA caused phenotypic differences among *OsANN4*-RNAi plants, and the other plants were eliminated with additional LaCL_3_ (Fig. 6b, c). The above results indicate that internal Ca^2+^ transport plays an important role in the response to ABA in rice, and that *OsANN4* participates in ABA-induced Ca^2+^ influx.

### OsANN4 interacts with the protein kinase OsCDPK24

**Fig. 7:**
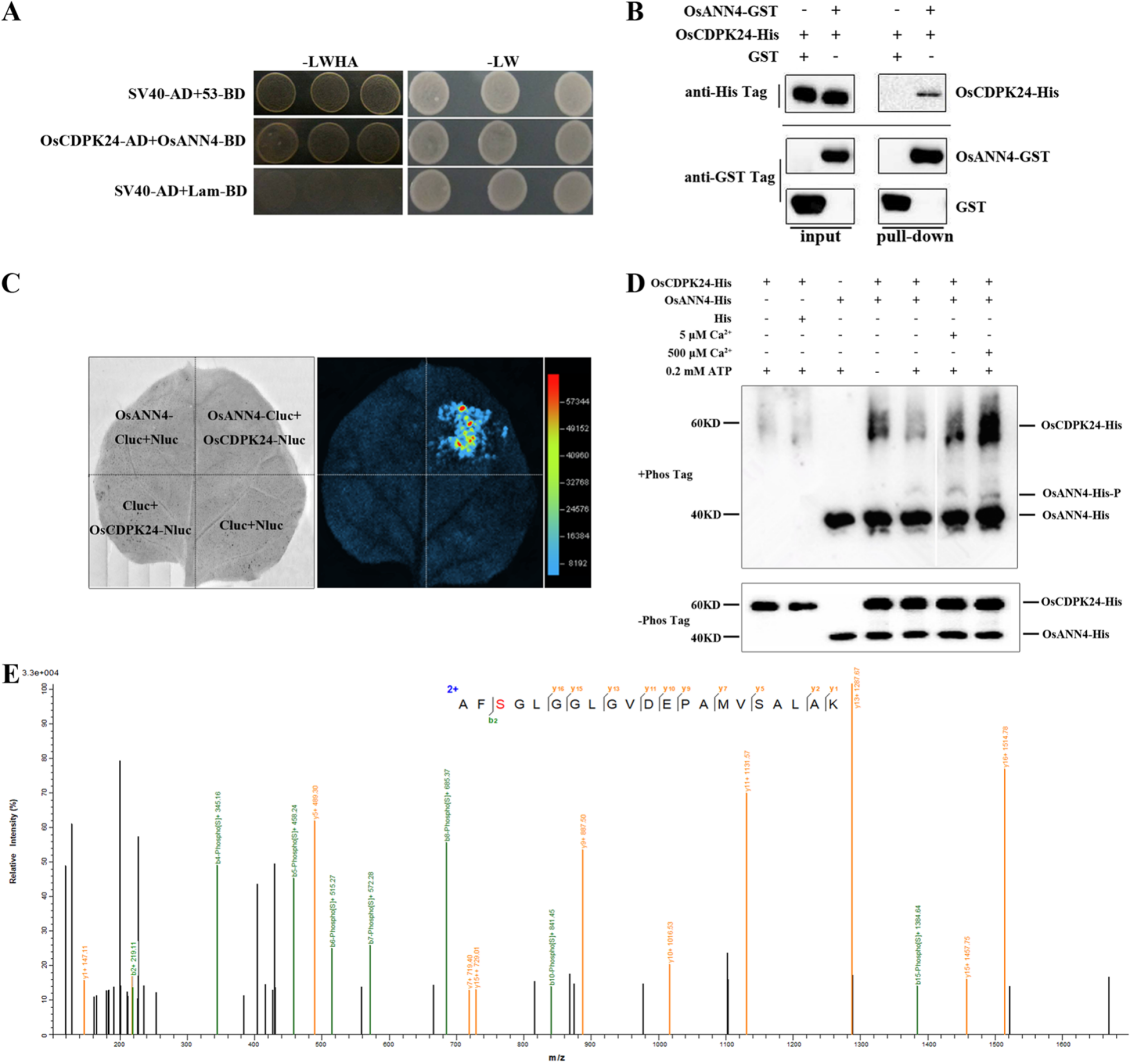
OsANN4 can interact with and be phosphorylated by the protein kinase OsCDPK24. **a** A yeast two-hybrid system was used to detect the interaction between OsANN4 and OsCDPK24. Yeast cells containing the indicated constructs were grown on dropout medium without Leu and Trp (-LW, right panel) and without Leu, Trp, His and Ade (-LWHA, left panel). **b** A GST pull-down assay was used to detect the interaction between OsANN4 and OsCDPK24. OsCDPK24-His protein was incubated with OsANN4-GST or GST protein. Pull-down proteins were detected by GST-tagged and His-tagged antibodies. **c** Firefly luciferase complementation imaging (LCI) assay detecting the interaction between OsANN4 and OsCDPK24. The colored scale bar indicates the luminescence intensity. **d** An in vitro kinase assay was performed with purified OsANN4-His and OsCDPK24-His using an SDS-PAGE gel (8%) containing a 50 μM Phos-tag. Signals were detected by using a His-tag antibody. **e** Mass spectrometry analysis of OsANN4 phosphorylation

In previous reports, annexins were shown to interact with protein kinases, including SAPKs and CDPKs [37, 48]. To further understand how *OsANN4* responds to ABA, we used a yeast two-hybrid assay to verify several potential rice protein kinase candidates, including Os01g0570500, Os10g0518800, Os01g0869900, and Os11g0171500. With the results, we did not find that Os01g0570500 and Os10g0518800 interacted with OsANN4 separately. However, Os01g0869900, which belongs to the SnRK2 family, showed a weak interaction with OsANN4. Furthermore, OsCDPK24 (Os11g0171500), a key regulator in response to ABA [55], showed a strong interaction with OsANN4 (Fig. 7a).

To further verify the interactions between OsANN4 and OsCDPK24, an in vitro pull-down system was carried out. OsCDPK24-His and OsANN4-GST recombinant proteins were induced in *Escherichia coli* and purified to perform the pull-down assay. OsANN4-GST pulled down OsCDPK24-His, whereas GST could not, further proving the interaction between OsANN4 and OsCDPK24 (Fig. 7b). We also obtained additional confirmation of the interaction between OsANN4 and OsCDPK24 in *Nicotianana benthamiana* leaves by using a luciferase complementation imaging (LCI) assay. After spraying D-luciferin to tobacco leaves, a fluorescent signal occurred when p*OsANN4*-Cluc and p*OsCDPK24*-Nluc were present simultaneously, which showed that OsANN4 can interact with OsCDPK24 (Fig. 7c).

### OsANN4 is phosphorylated by OsCDPK24

To understand the mechanism underlying the interaction of OsANN4 and OsCDPK24, we performed a phosphorylation assay in vitro to determine whether OsANN4 is a substrate of OsCDPK24. The Phos-tag reagent was used to separate phosphorylated proteins from nonphosphorylated proteins according to their different migration rates. When purified OsANN4-His and OsCDPK24-His were incubated together, phosphorylated OsANN4 bands were detectable with a His-tag antibody. The phosphorylation level of OsANN4 increased slightly after the addition of 5 μM and 500 μM Ca^2+^ (Fig. 7d). These results indicated that OsANN4 can be phosphorylated by OsCDPK24 and that the calcium signal can promote the phosphorylation process of OsANN4 by OsCDPK24.

**Fig. 8:**
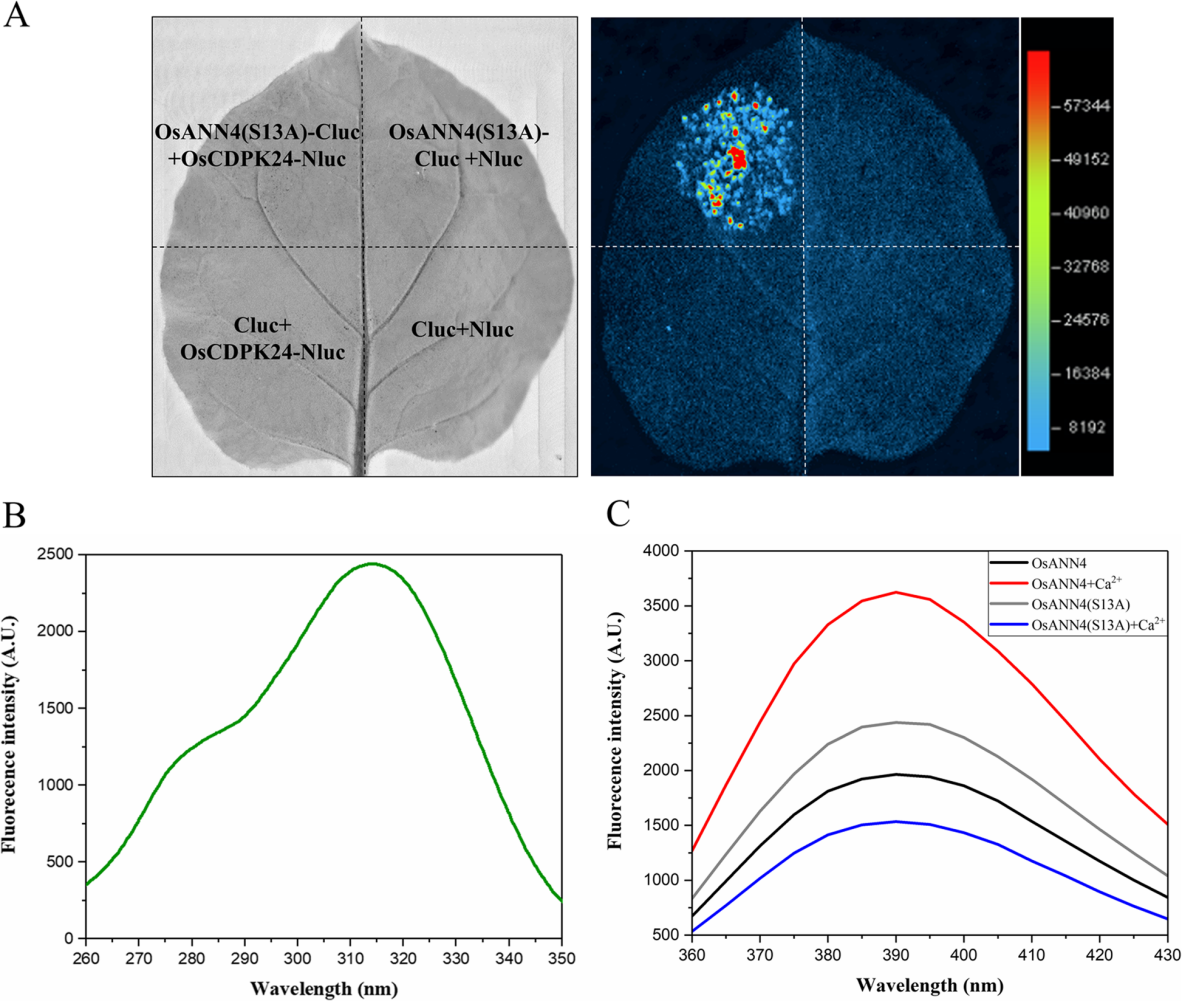
The protein properties of the mutant OsANN4 may be changed. **a** Firefly luciferase complementation imaging (LCI) assay detecting the interaction between OsANN4(S13A) and OsCDPK24. The colored scale bar indicates the luminescence intensity. **b** Fluorescence excitation of the purified OsANN4(S13A)-His protein. **c** Emission spectra of the purified OsANN4-His and OsANN4(S13A)-His proteins

To further analyze the exact site of phosphorylation in OsANN4, mass spectrometry was performed when OsCDPK24 was present. Mass spectrometry results suggested that OsANN4 can be phosphorylated by OsCDPK24, and the OsANN4 phosphorylation site was the 13th amino acid, which is a serine (Fig. 7e). Next, to inhibit the phosphorylation of OsANN4, we replaced the serine (S) residue with nonphosphorylatable alanine (A), named OsANN4 (S13A), and constructed the p*OsANN4 (S13A)*-Cluc vector for an LCI assay. The fluorescent signal was still detected when p*OsANN4(S13A)*-Cluc and p*OsCDPK24*-Nluc were present simultaneously (Fig. 8a), which suggested that the mutation of the phosphorylation site may not affect the interaction between OsANN4 and OsCDPK24.

Liu et al. demonstrated that the Ca^2+^ transport activity of AtANN1 can be enhanced by its phosphorylation of OST1 [42]. To examine whether the phosphorylation site affects its binding to Ca^2+^, the OsANN4(S13A)-His vector was constructed, and OsANN4(S13A)-His recombinant protein was induced and purified to perform the above fluorescence assay. Upon excitation at 315 nm (Fig. 8b), the same fluorescence emission spectra of OsANN4(S13A)-His and OsANN4 were observed; however, the fluorescence intensities were different (Fig. 8c). The results indicated that mutation of the phosphorylation site may change the conformation of OsANN4. After adding Ca^2+^, the fluorescence intensity of OsANN4(S13A)-His changed significantly, indicating that OsANN4(S13A)-His still has Ca^2+^ binding ability. In contrast to the obvious increase in the fluorescence intensity of OsANN4, the fluorescence intensity of OsANN4(S13A)-His was significantly reduced after adding Ca^2+^ (Fig. 8c), which implied that mutation of the phosphorylation site may affect the conformation of OsANN4 and further resulted in changing the binding activity with Ca^2+^.

## Discussion

### *OsANN4* is involved in ABA-induced antioxidant defense

As a stress plant hormone, ABA can trigger the accumulation of H_2_O_2_, thereby activating the ROS signaling system [1, 56, 57]. Excessive accumulation of ROS can cause toxic effects on proteins, lipids and nucleic acids, and the antioxidant defense system consisting of enzymatic and nonenzymatic antioxidants is essential for scavenging excess ROS [17, 18]. Evidence has shown that plant annexins have peroxidase activity and respond to abiotic stress by regulating the production of ROS [30, 37, 38, 45]. *OsANN1*-overexpressing plants eliminated excess ROS by increasing peroxidase activity, thereby improving the tolerance of rice to heat stress [37]. Under drought and high salinity, the activities of antioxidant enzymes in the *SpANN2*-overexpressing plants were higher than those in the WT plants, which contributed to improving the tolerance of transgenic tomato to drought and salt stresses [58]. Herein, we report that *OsANN4*, an ortholog of *AtANN4*, modulates H_2_O_2_ accumulation when exogenous ABA is applied. The expression of *OsANN4* was upregulated in rice in response to ABA (Fig. 1c, d), and knocking down *OsANN4* expression slowed the growth of rice with ABA treatment (Fig. 2 and Figure S2). Furthermore, the activities of SOD and CAT in *OsANN4*-RNAi plants were significantly lower than those in WT plants under ABA application (Fig. 3a, b). Consistent with the peroxidase activity results, the O_2_^.-^ and H_2_O_2_ contents in *OsANN4*-RNAi rice leaves were significantly higher than those in WT or *OsANN4*-OE leaves (Fig. 3c-e). We speculate that OsANN4 responds to exogenous ABA at least in part by regulating ROS and redox homeostasis.

### OsANN4 contributes to ABA-induced Ca^2+^ influx

In addition to having peroxidase activity, plant annexins also have Ca^2+^-binding activity, which is pivotal in plant responses to stress [30, 38]. Annexins are traditionally perceived as Ca^2+^-dependent phospholipid-binding proteins, which usually contain a characteristic type II Ca^2+^-binding residue in each corresponding repeat in vertebrates. However, in plant annexins, type II Ca^2+^-binding residues are absent in repeats 2 and 3 [59]. Although absent type II Ca^2+^-binding residues were found in OsANN4 (predicted at ScanProsite http://ca.expasy.org/tools/scanprosite/), our results still showed that OsANN4 has Ca^2+^-binding activity (Fig. 4a, b), which could be perceived as evidence that annexins can also bind to Ca^2+^ in an unknown and intricate manner.

Calcium is a key ion that controls a variety of cell activities to regulate different stimuli responses [60]. External stimuli cause Ca^2+^ flux to generate a specific Ca^2+^ signal, and the flux of Ca^2+^ mainly depends on Ca^2+^ channels, pumps (ATPase) and exchangers [61, 62]. In addition, it also relies on some nonspecifically transported Ca^2+^ proteins, such as cyclic nucleotide gated channels located on the plasma membrane and inner membrane, glutamate receptor homologs and annexins [31, 63]. Previous studies have shown that AtANN1 is involved in Ca^2+^ transport induced by multiple stresses, such as oxidative stress, drought, high salt, and heat [39, 43, 44, 54]. A recent study demonstrated that AtANN1 also regulated the increase in [Ca^2+^]_cyt_ induced by cold stress [42]. In addition, the functions of AtANN4, GhANN8b and OsANN10 in the stress response were all reported to be related to Ca^2+^ [38, 50, 51]. In the current study, we found that OsANN4 was located at the cell periphery and had Ca^2+^-binding properties (Fig. 4), suggesting that OsANN4 may modulate Ca^2+^ transport in response to exogenous ABA.

Subsequently, we used NMT technology to detect the net Ca^2+^ flux with or without exogenous ABA treatment. The data showed that in the absence of ABA, there was weak Ca^2+^ efflux in the root tips of WT plants, and there was Ca^2+^ influx in the root tips of *OsANN4* transgenic plants (Fig. 5a). After adding ABA, the Ca^2+^ influx rate of WT and *OsANN4*-OE rice root tips increased significantly, while the Ca^2+^ influx rate in the root tips of *OsANN4*-RNAi plants did not change significantly (Fig. 5b, c). This result indicates that the addition of exogenous ABA induces Ca^2+^ influx, and its purpose may be to increase the [Ca^2+^]_cyt_ to correspond to exogenous ABA. The downregulated expression of *OsANN4* in rice weakened ABA-induced Ca^2+^ influx to a certain extent, indicating that OsANN4 may be involved in ABA-induced Ca^2+^ influx. In addition, in the presence of ABA, the difference between *OsANN4* knockdown plants and WT and *OsANN4* overexpression plants was alleviated to a certain extent after adding LaCl_3_ (Fig. 6 and Figure S3), which further verified our speculation. After adding ABA, although the Ca^2+^ influx in the root tips of *OsANN4* knockdown plants did not increase significantly, they still showed a state of influx (Fig. 5). There are ten putative annexins in the rice genome. At present, both OsANN1 and OsANN10 have been shown to have Ca^2+^ binding ability, and OsANN10 may mediate Ca^2+^ transport induced by osmotic stress. Therefore, we believe that other annexins or Ca^2+^ transporters are also involved in ABA-induced Ca^2+^ transport.

### OsANN4 functions with OsCDPK24 in response to ABA

CDPKs belong to the serine/threonine protein kinase family and can sense transient changes in Ca^2+^ in the cytoplasm [64]. Growing evidence shows that CDPKs play an important role in the response to abiotic stress and plant hormone signaling pathways. For instance, a previous study showed that OsCDPK12 can induce the expression of the antioxidant genes *OsAPX2* and *OsAPX8* under salt stress and reduce the salt-induced accumulation of H_2_O_2_, suggesting that OsCDPK12 positively regulates ROS detoxification by controlling the expression of antioxidant genes [65]. AtCPK6 positively modulated ABA signaling and drought response by phosphorylating ABF3 and ABI5 [66]. OsCDPK14 is involved in the regulation of ABA signaling at least in part by interacting with OsDi19-4 and phosphorylating OsDi19-4 [55]. In ABA signaling, ZmCPK11 acts upstream of *ZmMPK5* and participates in ABA-induced antioxidant defense [67].

Studies have shown that the phosphorylation modification of annexin plays a role in signaling. For example, in ABA signaling, AtANN1 can be phosphorylated by AtSnRK2s, and in response to cold stress, AtANN1 can be phosphorylated by OST1/SnRK2.6 [42, 49]. AtANN4 participates in the salt stress response by being phosphorylated by SOS2 [50]. Cotton annexin GhANN8b can be dephosphorylated by GhDsPTP3a and participates in the response to salt stress [51]. However, there have been few studies on the relationship between plant annexins and CDPKs as calcium ion sensors. In this study, we demonstrate that OsANN4 interacts with OsCDPK24 and is a substrate of OsCDPK24, and the phosphorylation site of OsANN4 is the 13th serine, which is a key site for phosphorylation (Fig. 7). Although OsANN4 with the 13th serine mutated to alanine can still interact with OsCDPK24, the conformation of OsANN4 might be changed, and further resulted in changing its binding activity with Ca^2+^ (Fig. 8c). However, the mechanism concerning how the interaction between OsANN4 and OsCDPK24 regulates response to ABA and Ca^2+^ require further study.

## Conclusions

In this study, the calcium-binding protein OsANN4 was identified in rice. OsANN4 has the ability to maintain redox balance and is involved in the response to ABA. The phosphorylation of OsANN4 by OsCDPK24 might play key role in contribution to respond to ABA signaling.

## Methods

### Vector construction for recombinant protein expression

To obtain full-length OsANN1 cDNA encoding, total RNA was isolated from 7-d-old rice seedlings, and specific primers were designed based on the sequence of *OsANN4*. The specific PCR products were cloned into the p1301-HA, pMDC83, pTCK303, pET28a, pGEX4T-1, pCAMBIA-Cluc and pGBKT7 vectors to generate Ubi::OsANN4-HA, 35S::OsANN4-GFP, OsANN4-RNAi, OsANN4-His, OsANN4-GST, OsANN4-Cluc, and OsANN4-BD constructs, respectively. Site-directed mutagenesis of *OsANN4* was carried out by using a fast mutagenesis kit (Fast Site-Directed Mutagenesis Kit, Tiangen, China). To construct the *OsCDPK24* expression vector, specific primers based on the sequence of *OsCDPK24* were used, and the PCR products were cloned into the pET28a, pCAMBIA-Nluc and pGADT7 vectors to generate OsCDPK24-His, OsCDPK24-Nluc, and OsCDPK24-AD constructs, respectively. All primers used are listed in Supplementary Table S1.

### Plant materials and ABA treatment

The rice cultivar Nipponbare was provided by the China National Rice Research Institute. Nipponbare rice seeds were used as original plants and the WT control in this study. We constructed a series of rice plants consisting of knockdown or overexpression as well as others that mediated *Agrobacterium* transformation [68]. Rice seeds were surface-sterilized with 50% NaClO for 20 min, rinsed 10 times with sterile distilled water and then grown on 1/2 MS medium. The rice plants were grown in a standard culture solution in a greenhouse with a light/dark cycle of 16/8 h and 50% relative humidity at 28/25°C (day/night). For ABA treatment, the seeds were planted on 1/2 MS medium supplemented with 10 μM or 20 μM ABA for 18 d. Control seeds were planted on 1/2 MS medium and cultured with water after 18 d.

### RNA isolation, RT-PCR, and quantitative RT-PCR analysis

Total RNA was isolated from different tissues of rice plants with RNAiso plus reagent (TaKaRa, Japan). Purified RNA (2 μg) was incubated with DNase 1 (RNase-Free DNase, Thermo Fisher, USA) at 37°C for 30 min. First-strand cDNA was synthesized using the PrimeScript™ First Strand cDNA Synthesis Kit (TaKaRa, Japan) to perform RT-PCR. One microgram of purified total RNA was used to obtain first-strand cDNA with the PrimeScript™ RT reagent Kit with gDNA Eraser (TaKaRa, Japan), and qRT-PCR was performed with specific primers *OsANN4* and *OsACTIN1* (see Supplementary Table S1) using a C1000 Real-Time PCR instrument (Bio-Rad, USA) and SYBR ® Premix Ex Taq™ II (TaKaRa, Japan). *OsACTIN1* (Os03g0718100) was used as an internal control for the normalization of all data in this experiment. Three independent biological replicates were assayed.

### Superoxide dismutase (SOD) and catalase (CAT) activity assays

Seven-day-old seedlings were homogenized in 1 mL extraction buffer [50 mM phosphate, 1 mM EDTA-Na_2_, 1% (w/v) polyvinyl pyrrolidone, pH 7.4], and the homogenate was centrifuged at 8,000 g for 30 min at 4°C. The supernatant was used for further assays. Soluble protein contents were examined by the Bradford method with BSA as a standard control. The activities of SOD (EC1.15.1.1) and CAT (EC1.11.1.6) were tested as described by Jiang and Zhang [69].

### Detection of H_2_O_2_ content

The H_2_O_2_ content was examined using a hydrogen peroxide assay kit (Beyotime Biotechnology, Shanghai, China), as described by Zafar et al. (2020) with some modifications [70, 71]. Briefly, the leaves of 7-d-old seedlings (0.01 g, Fw) were homogenized in 200 μL lysis solution and centrifuged at 8,000 g for 30 min at 4°C. Fifty microliters of supernatant and 100 μL of hydrogen peroxide detection reagent were added to the detection wells and incubated at room temperature (25°C) for 30 min, and then the A560 was immediately determined. The concentration of H_2_O_2_ in the sample was calculated according to the standard curve.

### In situ detection of O_2_^·–^ or H_2_O_2_

The in situ detection of O_2_^.-^ and H_2_O_2_ was carried out according to Bei et al. (2015) with some modifications [37]. To detect O_2_^.-^ in situ, 7-d-old plant leaves were detached and immersed in 6 mM NBT, vacuumed for 30 min, and then placed under light for 8 h at 25°C. To remove the chlorophyll and reveal the dark blue blots, the leaves were placed into a decolorizing buffer (ethanol: ethylic acid: glycerol=3:1:1) until the chlorophyll was completely removed. To detect H_2_O_2_ in situ, 7-d-old plant leaves were detached and immersed in 1 mg/mL DAB solution (pH 3.8), vacuumed for 30 min, and then placed in the dark at 25°C for 8 h. After the leaves were bleached by the decolorizing solution, the brown spots were the result of the reaction between DAB and H_2_O_2_.

### Subcellular localization of OsANN4

For subcellular localization analyses, the *OsANN4* coding region was fused to the N-terminus of GFP using the PMDC83 backbone to construct CaMV35S::OsANN4-GFP. The fusion construct was introduced into *Agrobacterium* EHA105 cells and then transformed into rice calli as previously described [72]. The OsANN4-GFP protein signal was observed using confocal laser-scanning microscopy (Zeiss LSM710, Germany).

### Fluorescence measurements of OsANN4

This experiment was performed according to the method described in a previous study [37]. The assay media contained 2 μM recombinant OsANN4 protein and 0 mM or 2 mM Ca^2+^, and fluorescence spectroscopy was carried out by using a fluorescence spectrophotometer (F-4600; Hitachi, Japan).

### Measurements of net Ca^2+^ flux with non-invasive micro-test technology

The net Ca^2+^ flux was measured using Non-invasive Micro-test Technology at Xuyue (Beijing) Sci. & Tech. Co., Ltd., Beijing, China. The root tips of 3-d-old seedlings with or without ABA treatment were washed and transferred to measurement buffer (0.1 mM KCl, 0.1 mM CaCl_2_, 0.1 mM MgCl_2_, 0.5 mM NaCl, 0.3 mM MES, 0.2 mM Na_2_SO4, pH 6.0) for 30 min equilibration, and then the net Ca^2+^ flux was recorded within 5 min. All samples in the test were repeated at least three times.

### Yeast two-hybrid analysis

The yeast two-hybrid assays were carried out as described [37]. In brief, the open reading frames of OsCDPK24 and OsANN4 were independently cloned into the expression vector pGADT7 (AD) or pGBKT7 (BD). The construct pairs were cotransformed into AH109 yeast cells, and the transformed yeast cells were plated on SD/-Leu/-Trp media (-LW). After growing for 3 d, the clones were transferred to SD/-Leu/-Trp/-His/-Ade (-LWHA) or -LW media for 3–5 d. A positive control interaction between the 53 protein and SV40 protein and a negative control interaction between the Lam protein and SV40 protein were observed.

### Pull-down assay

The *E. coli* Rosetta strain containing pET28a-*OsCDPK24*, pGEX4T-1-OsANN4 or pGEX4T-1 was induced at 18°C overnight, the expressed OsCDPK24-His protein was purified using Ni-NTA Resin (Sangon Biotech, China), and OsANN4-GST or GST protein was purified using glutathione sepharose 4B beads (GE). Supernatants containing GST or OsANN4-GST were incubated with OsCDPK24-His in 0.5 ml of interaction buffer (25 mM Tris pH 7.2, 150 mM NaCl) overnight at 4°C. GST beads were added to the protein mixture and incubated for 2 h on a rotating wheel at 4°C followed by washing five times with wash buffer (25 mM Tris pH 7.2, 150 mM NaCl, 0.1% NP-40). Then, protein retained on the beads was separated on SDS-PAGE gel and analyzed by anti-His antibody.

### Luciferase complementation imaging (LCI) assay

A luciferase complementation imaging assay was carried out as described [70]. Briefly, the *OsANN4* and *OsCDPK24* coding regions were cloned into the pCAMBIA-Cluc and pCAMBIA-Nluc vector, respectively, and then transformed into *Agrobacterium* strain GV3101. *Agrobacterium* transformants containing p*OsANN4*-Cluc, p*OsCDPK24*-Nluc, pCAMBIA-Cluc and pCAMBIA-Nluc were adjusted to OD_600_=0.5–0.6 and then paired and injected into tobacco leaves. After spraying the tobacco leaves with 2.5 mM D-luciferin (Goldbio, USA), fluorescent signals were detected and photographed post infiltration by using a Fusion FX7 (Vilber, France) imaging system.

### In vitro kinase assay

An in vitro kinase assay was performed as described with minor modifications [55]. In brief, purified OsANN4-His (5 μg) was incubated with purified OsCDPK24-His (1.5 μg) in kinase buffer (20 mM Tris-HCl (pH 7.5), 10 mM MgCl_2_, 100 mM NaCl, 1 mM DTT) containing 2 mM ATP at 30°C for 10 h, and the reactions were terminated by boiling with 6× SDS loading buffer. Samples were separated by 8% Phos-tag SDS-PAGE gel containing 50 mM Phos-Tag (APExbio, China) and 100 mM MnCl_2_ and then transferred to nitrocellulose membranes. The signals were detected with an anti-His antibody (CWbio, China). Mass spectrometry to detect phosphorylation was finished by Applied Protein Technology Co., Ltd. (Shanghai, China).

## Supplementary Information

**Additional file 1: Figure S1.** Identification of *OsANN4* transgenic plants at the transcriptional level. **a.** Relative expression of *OsANN4* in *OsANN4*-OE transgenic rice. **b.** Relative expression of *OsANN4* in *OsANN4*-RNAi transgenic rice. Values represent the means ± SD from three independent repeats, and different letters indicate significant differences (one-way ANOVA, *P*<0.05).

**Additional file 2: Figure S2.** Analysis of germination and rooting rate. **a.** ABA responses of WT and *OsANN4* transgenic lines during seed germination. The photos were taken 3 d post germination. **b.** Analysis of rooting rate with 0 μM ABA treatment. **c.** Analysis of rooting rate with 10 μM ABA treatment. **d.** Analysis of rooting rate with 20 μM ABA treatment. Values represent means ± SD from three independent repeats.

**Additional file 3: Figure S3.** Analysis of germination and rooting rate in the presence of LaCl_3_. **a.** ABA responses of WT and *OsANN4* transgenic lines in the presence of LaCl_3_ during seed germination. The photos were taken 1 d post germination. **b.** Analysis of rooting rate under 0 μM ABA treatment in the presence of LaCl_3_. **c.** Analysis of rooting rate under 10 μM ABA treatment in the presence of LaCl_3_. **d.** Analysis of rooting rate under 20 μM ABA treatment in the presence of LaCl_3_. Values represent means ± SD from three independent repeats.

**Additional file 4: Table S1.** Primer sequences for plasmid construction and qRT-PCR.

**Additional file 5: Figure S4.** Full length image of the western blots shown in Fig. 7B. Immunoblot analysis of GST (≈27 KDa) in both input and pull-down proteins.

**Additional file 6: Figure S5.** Full length image of the western blots shown in Fig. 7B. Immunoblot analysis of OsANN4-GST (≈63 KDa) in both input and pull-down proteins.

**Additional file 7: Figure S6.** Full length image of the western blots shown in Fig. 7B. Immunoblot analysis of OsCDPK24-His (≈60 KDa) in input proteins with His antibody.

**Additional file 8: Figure S7.** Full length image of the western blot shown in Fig. 7B. Immunoblot analysis of OsCDPK24-His (≈60 KDa) in pull-down proteins with His antibody.

**Additional file 9: Figure S8.** Full length image of the western blot shown in Fig. 7B. Immunoblot analysis was performed with purified OsANN4-His and OsCDPK24-His using an SDS-PAGE gel (8%) containing a 50 μM Phos-tag.

**Additional file 10: Figure S9.** Full length image of the western blot shown in Fig. 7B. Immunoblot analysis was performed with purified OsANN4-His and OsCDPK24-His using an SDS-PAGE gel (8%).

## Data Availability

The datasets and material used and analyzed in this study are available from the corresponding authors on reasonable request. This research doesn’t contain any omics data. All the genes were discovered in Rice Genome Annotation Project (http://rice.plantbiology.msu.edu/index.shtml) as follows: *OsANN4*(LOC_Os05g31750), *OsCDPK24* (LOC_Os11g07040), *OsACTIN1* (LOC_Os03g50885).

## Abbreviations

ABA: Abscisic acid
SOD: Superoxide dismutase
CAT: Catalase
NMT: Non-invasive Micro-testing Technology
START: Steroidogenic regulatory protein (StAR)-related lipid-transfer
PYR1: Pyrabactin resistance 1
PYL1-PYL13: PYR1-like 1-13
PP2Cs: Phosphatase 2Cs
SnRK2s: Suc nonfermenting-1-related protein kinase 2
ROS: Reactive oxygen species
APX: Ascorbate peroxidase
GR: Glutathione reductase
[Ca^2+^]_cyt_: Cytosolic free Ca^2+^ concentration
CDPKs: Ca^2+^-dependent protein kinases
CBL/CIPKs: Calcineurin B-like proteins/CBL interacting protein kinases
OST1/SnRK2.6: Protein kinase open stomatal 1
DAB: 3,3’-diaminobenzidine
NBT: Nitro-blue tetrazolium
