# Supplementary figures and images for: OsANN4 modulates ROS production and mediates Ca2+ influx in response to ABA
Source: BMC Plant Biol. 2021 Oct 18;21:474. doi: 10.1186/s12870-021-03248-3 (PMC8522085; doi:10.1186/s12870-021-03248-3)

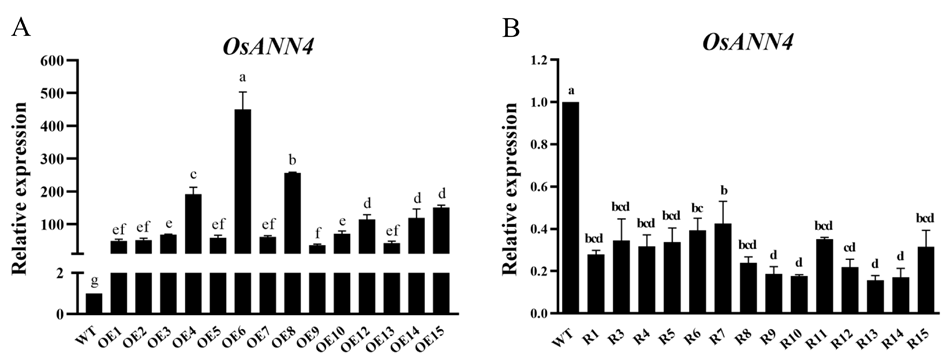

Supplement: Supplementary file 1 — Additional file 1: Figure S1. Identification of OsANN4 transgenic plants at the transcriptional level. a. Relative expression of OsANN4 in OsANN4-OE transgenic rice. b. Relative expression of OsANN4 in OsANN4-RNAi transgenic rice. Values represent the means ± SD from three independent repeats, and different letters indicate significant differences (one-way ANOVA, P<0.05). [file 12870_2021_3248_MOESM1_ESM.tif]

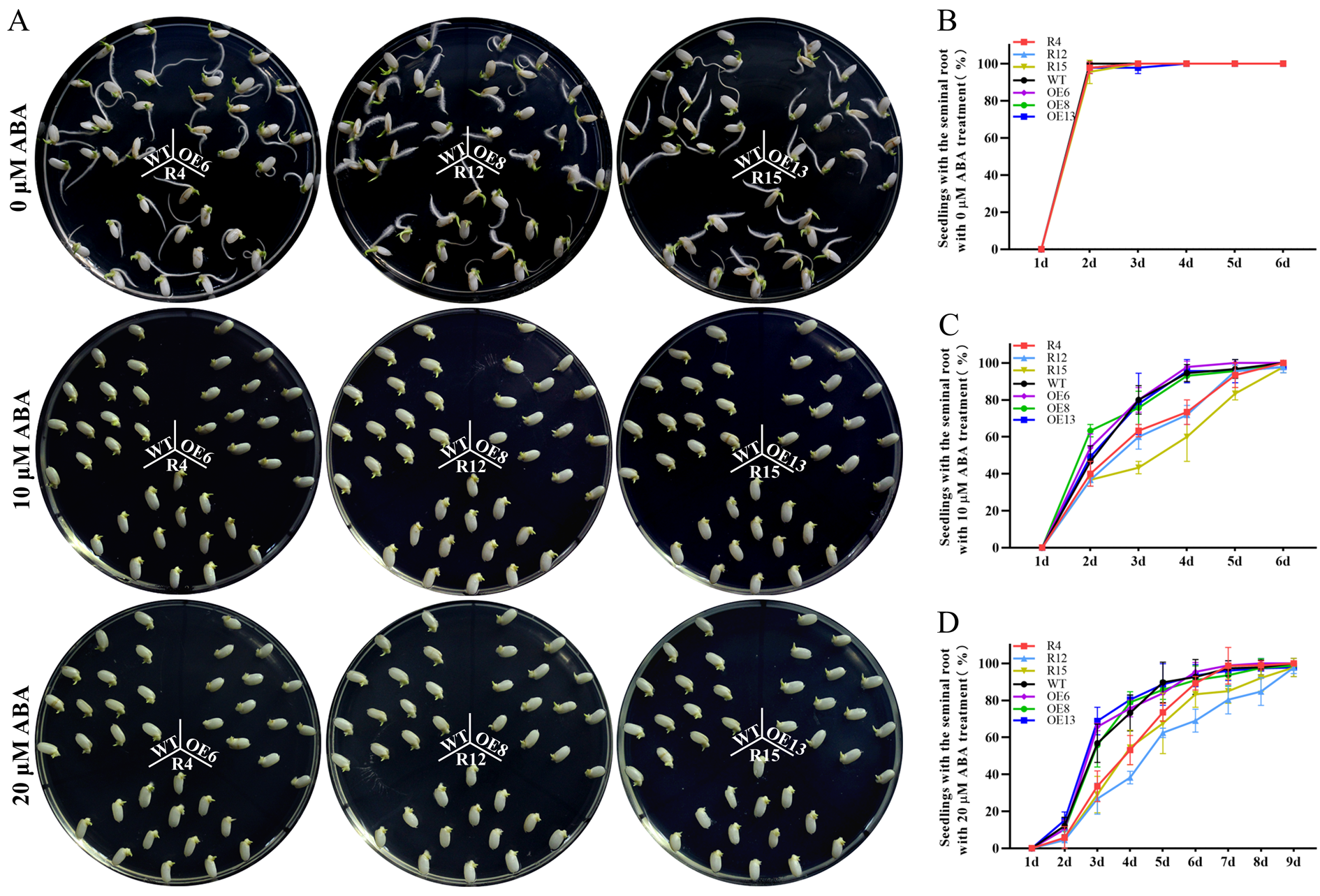

Supplement: Supplementary file 2 — Additional file 2: Figure S2. Analysis of germination and rooting rate. a. ABA responses of WT and OsANN4 transgenic lines during seed germination. The photos were taken 3 d post germination. b. Analysis of rooting rate with 0 μM ABA treatment. c. Analysis of rooting rate with 10 μM ABA treatment. d. Analysis of rooting rate with 20 μM ABA treatment. Values represent means ± SD from three independent repeats. [file 12870_2021_3248_MOESM2_ESM.tif]

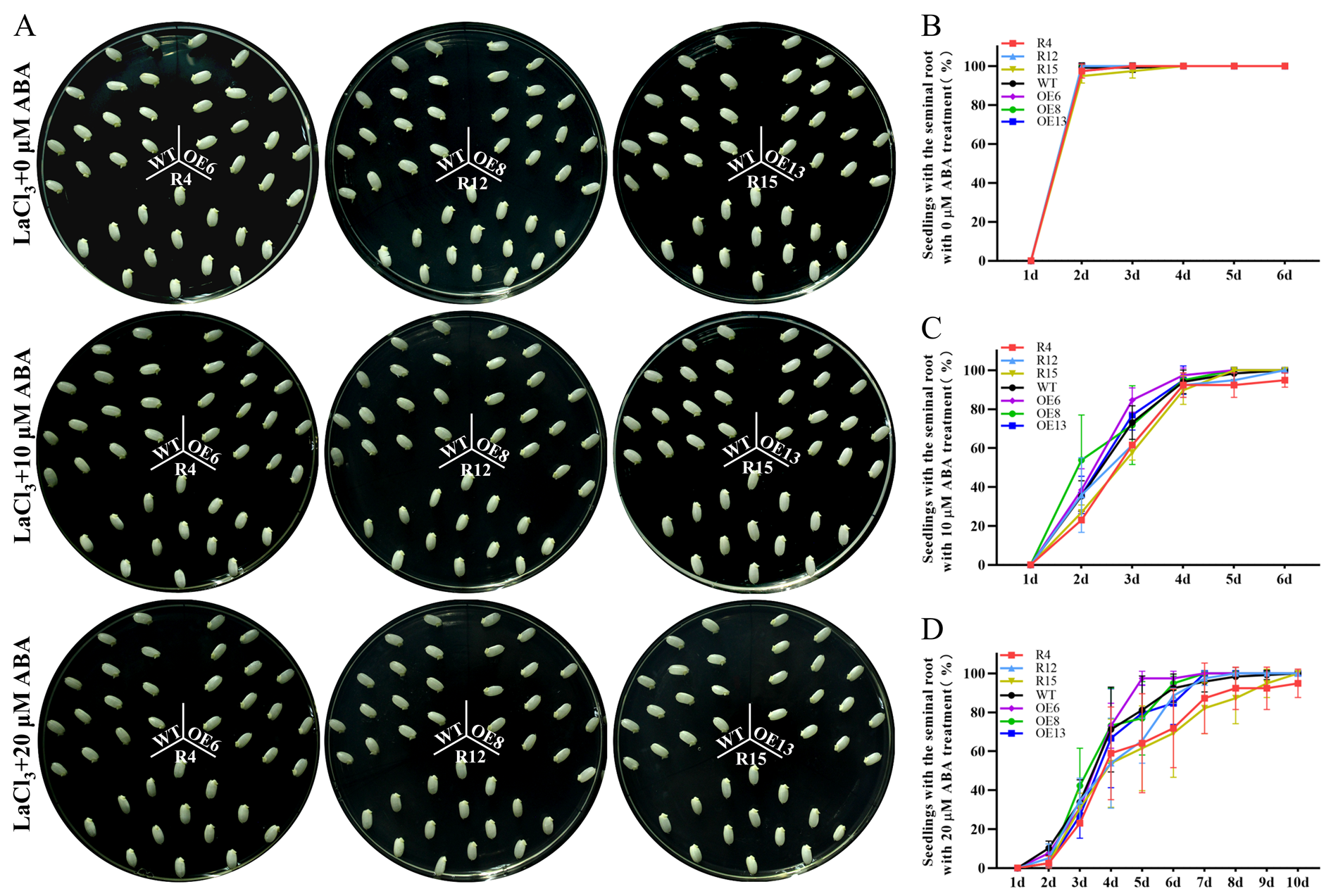

Supplement: Supplementary file 3 — Additional file 3: Figure S3. Analysis of germination and rooting rate in the presence of LaCl3. a. ABA responses of WT and OsANN4 transgenic lines in the presence of LaCl3 during seed germination. The photos were taken 1 d post germination. b. Analysis of rooting rate under 0 μM ABA treatment in the presence of LaCl3. c. Analysis of rooting rate under 10 μM ABA treatment in the presence of LaCl3. d. Analysis of rooting rate under 20 μM ABA treatment in the presence of LaCl3. Values represent means ± SD from three independent repeats. [file 12870_2021_3248_MOESM3_ESM.tif]

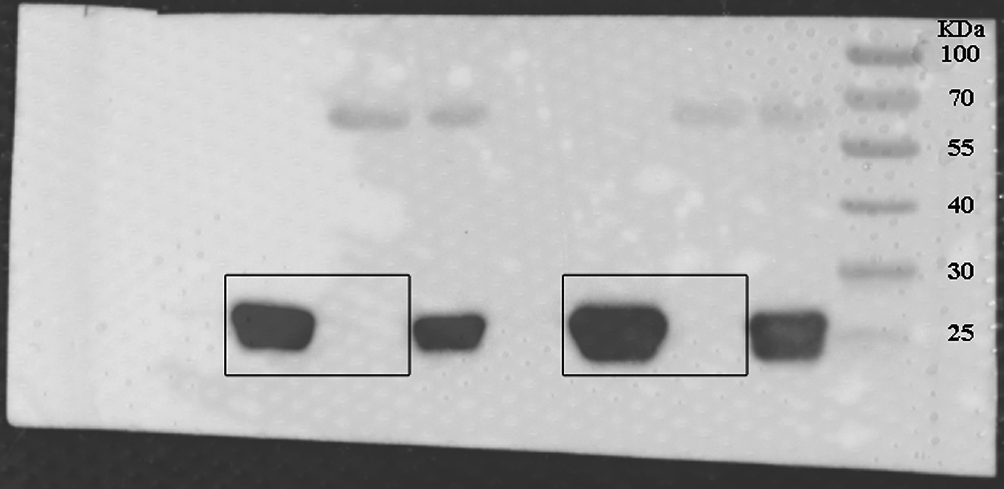

Supplement: Supplementary file 5 — Additional file 5: Figure S4. Full length image of the western blots shown in Fig. 7B. Immunoblot analysis of GST (≈27 KDa) in both input and pull-down proteins. [file 12870_2021_3248_MOESM5_ESM.tif]

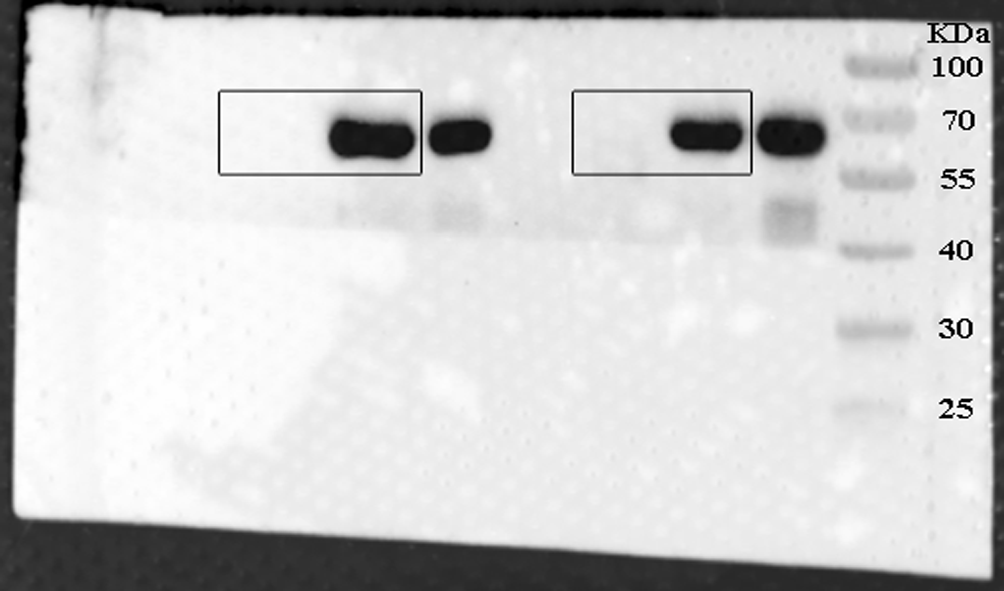

Supplement: Supplementary file 6 — Additional file 6: Figure S5. Full length image of the western blots shown in Fig. 7B. Immunoblot analysis of OsANN4-GST (≈63 KDa) in both input and pull-down proteins. [file 12870_2021_3248_MOESM6_ESM.tif]

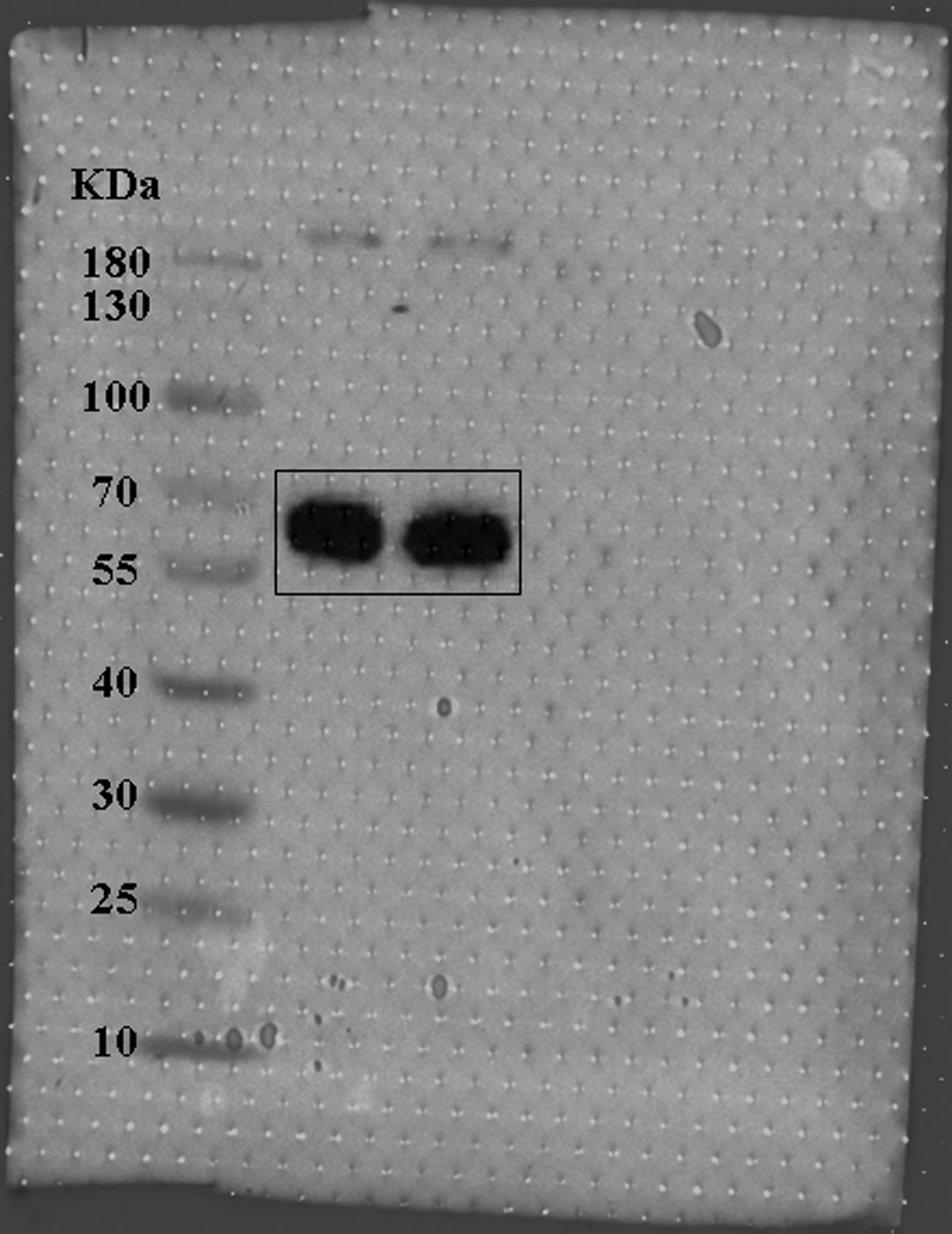

Supplement: Supplementary file 7 — Additional file 7: Figure S6. Full length image of the western blots shown in Fig. 7B. Immunoblot analysis of OsCDPK24-His (≈60 KDa) in input proteins with His antibody. [file 12870_2021_3248_MOESM7_ESM.tif]

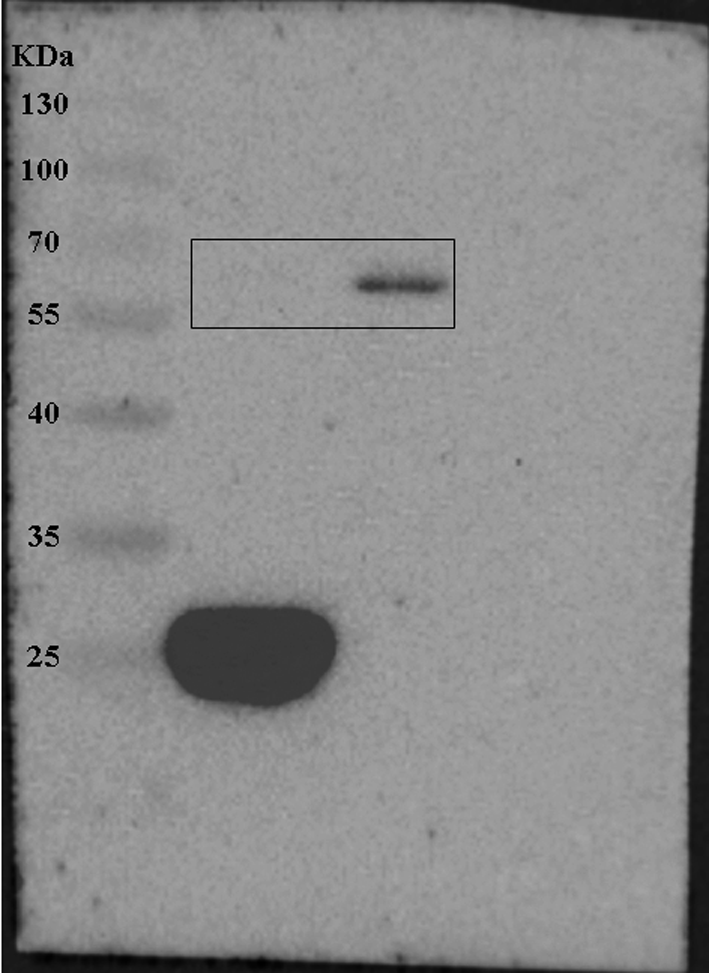

Supplement: Supplementary file 8 — Additional file 8: Figure S7. Full length image of the western blot shown in Fig. 7B. Immunoblot analysis of OsCDPK24-His (≈60 KDa) in pull-down proteins with His antibody. [file 12870_2021_3248_MOESM8_ESM.tif]

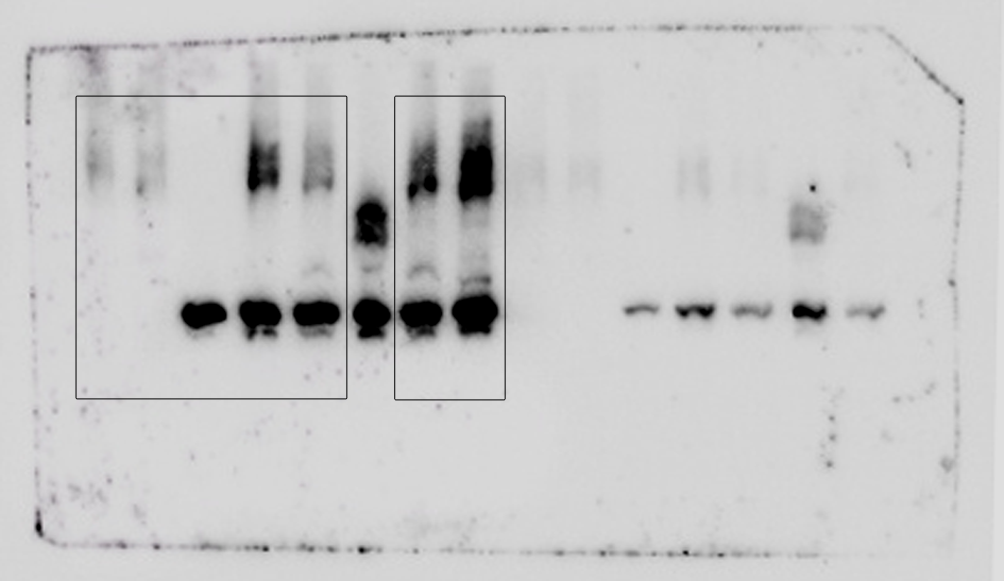

Supplement: Supplementary file 9 — Additional file 9: Figure S8. Full length image of the western blot shown in Fig. 7B. Immunoblot analysis was performed with purified OsANN4-His and OsCDPK24-His using an SDS-PAGE gel (8%) containing a 50 μM Phos-tag. [file 12870_2021_3248_MOESM9_ESM.tif]

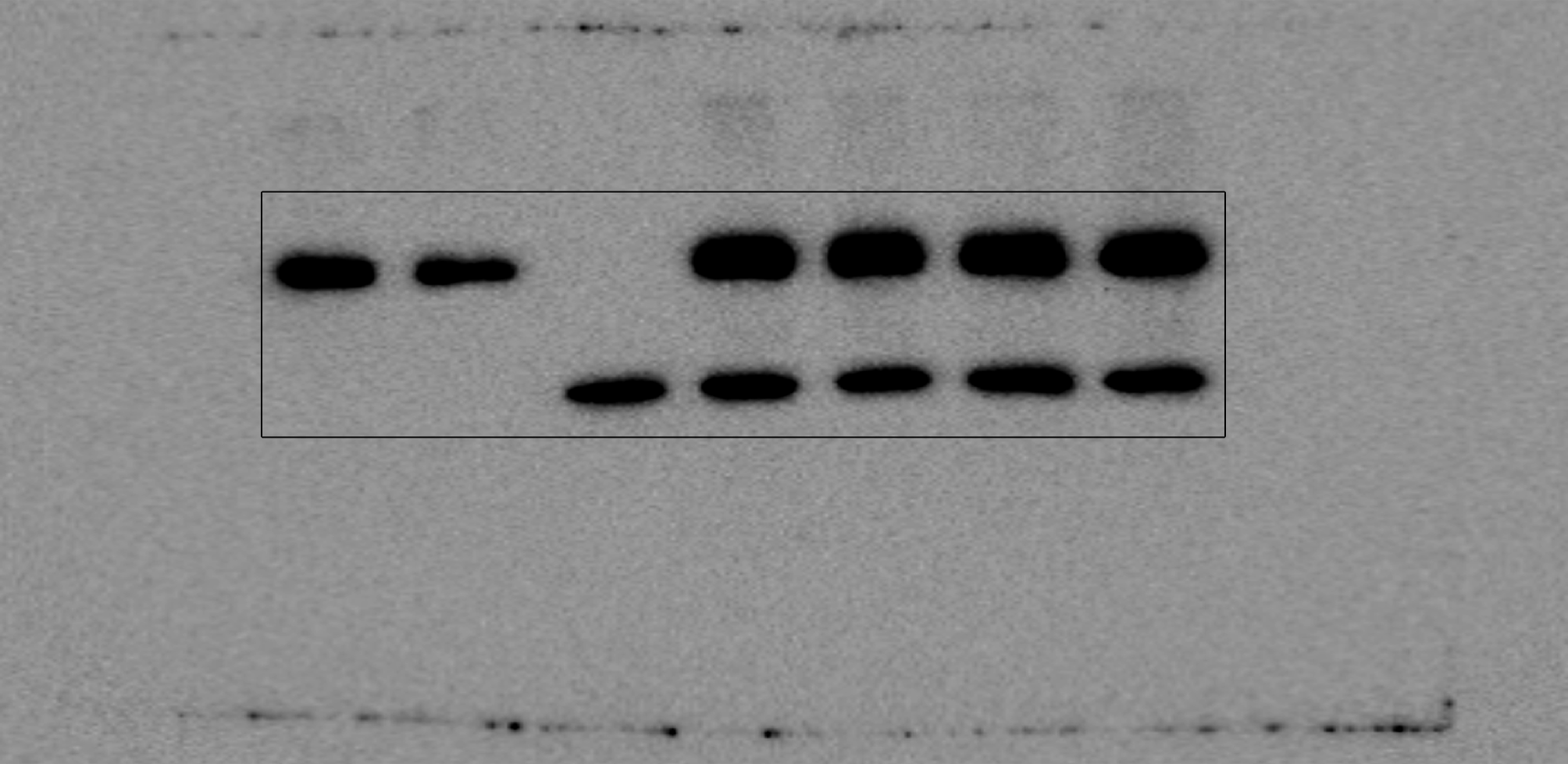

Supplement: Supplementary file 10 — Additional file 10: Figure S9. Full length image of the western blot shown in Fig. 7B. Immunoblot analysis was performed with purified OsANN4-His and OsCDPK24-His using an SDS-PAGE gel (8%). [file 12870_2021_3248_MOESM10_ESM.tif]
