# Supplementary material for: OsANN4 modulates ROS production and mediates Ca2+ influx in response to ABA
Source: BMC Plant Biol. 2021 Oct 18;21:474. doi: 10.1186/s12870-021-03248-3 (PMC8522085; doi:10.1186/s12870-021-03248-3)
Supplement: Supplementary file 4 — Additional file 4: Table S1. Primer sequences for plasmid construction and qRT-PCR. [file 12870_2021_3248_MOESM4_ESM.doc]

**Supplementary Table 1**

**Primer sequences used in plasmids construction and PCR**

| Primers | Sequence | Purpose |
| --- | --- | --- |
| P1 | 5’TTCTGCAGCGGGATCCATGGCTGATGAAATCCA3’ | *OsANN4* cDNA amplification for 1301-HA vector |
| P2 | 5’CGTAACGCGTGGATCCTTCTTGCCGCCGGC3’ |
|  |  |  |
| P3 | 5’TCTAGAATGGCTGATGAAATCCA3’ | *OsANN4* cDNA amplification for pMDC83 vector |
| P4 | 5’GGTACCCTTGCCGCCGGC3’ |
|  |  |  |
| P5 | 5’GGGGTACCACTAGTTCACCACCCGGAGCAAGC3’ | *OsANN4* RNAi fragment amplification |
| P6 | 5’CGGGATCCGAGCTCGGCGACGAGCGAGAGGAGC3’ |
|  |  |  |
| P7 | 5’GAATTCATGGCTGATGAAATCCA3’ | *OsANN4* cDNA amplification for prokaryotic expression |
| P8 | 5’AAGCTTTCACTTGCCGCC3’ |
|  |  |  |
| P9 | 5’AAGCTTGGATGCAGCCGGACCCGAGC3’ | *OsCDPK24* cDNA amplification for prokaryotic expression |
| P10 | 5’CTCGAGTTAGCTGTTGCTGGGGTTCAAGATC3’ |
|  |  |  |
| P11 | 5’GGTACCATGGCTGATGAAATCCAGCATCTGA3’ | *OsANN4* cDNA amplification for pCAMBIA-Cluc vector |
| P12 | 5’GTCGACTCACTTGCCGCCGGCGACGA3’ |
|  |  |  |
| P13 | 5’TCTAGAATGCAGCCGGACCCGAGC3’ | *OsCDPK24* cDNA amplification for pCAMBIA-Nluc vector |
| P14 | 5’CTCGAGGCTGTTGCTGGGGTTCAA3’ |
|  |  |  |
| P15 | 5’ CGGGTTCAGGAAGAGCTTCAAT3’ | Q-PCR( *OsANN4*) |
| P16 | 5’ CATCGCCCACATCACCATCA3’ |
|  |  |  |
| P17 | 5’CATCTCTCAGCACATTCCAGCAG3’ | Q-PCR( *OsACTIN1*) |
| P18 | 5’AGGAGGACGGCGATAACAGC3’ |
|  |  |  |
| P19 | 5’CATATGATGGCTGATGAAATCCA3’ | *OsANN4* cDNA amplification for pGBKT7 vector |
| P20 | 5’GAATTCTCACTTGCCGCC3’ |
|  |  |  |
| P21 | 5’CATATGATGCAGCCGGACCCGAGC3’ | *OsCDPK24* cDNA amplification for pGADT7 vector |
| P22 | 5’CTCGAGGCTGTTGCTGGGGTTCAA3’ |
